# Microcrystal electron diffraction structure of Toll-like receptor 2 TIR-domain-nucleated MyD88 TIR-domain higher-order assembly

**DOI:** 10.1107/S2059798324008210

**Published:** 2024-09-04

**Authors:** Y. Li, L. C. Pacoste, W. Gu, S. J. Thygesen, K. J. Stacey, T. Ve, B. Kobe, H. Xu, J. D. Nanson

**Affiliations:** a School of Chemistry and Molecular Biosciences The University of Queensland Brisbane Queensland4072 Australia; b Institute of Molecular Bioscience The University of Queensland Brisbane Queensland4072 Australia; c Australian Infectious Diseases Research Centre The University of Queensland Brisbane Queensland4072 Australia; d Department of Materials and Environmental Chemistry Stockholm University Frescativägen 8 114 18Stockholm Sweden; e Institute for Glycomics Griffith University Southport Queensland4215 Australia; f Gulbali Institute Charles Sturt University Wagga Wagga New South Wales2678 Australia; University of Melbourne, Australia

**Keywords:** MicroED, MyD88, TIR domains, Toll/interleukin-1 receptor protein, higher-order assemblies, Toll-like receptors, signalosomes

## Abstract

Eukaryotic TIR (Toll/interleukin-1 receptor protein) domains signal via TIR–TIR interactions, either by self-association or by interaction with other TIR domains. In mammals, TIR domains are found in Toll-like receptors (TLRs) and cytoplasmic adaptor proteins involved in pro-inflammatory signaling. Previous work revealed that the MAL TIR domain (MAL^TIR^) nucleates the assembly of MyD88^TIR^ into crystalline arrays *in vitro*. A microcrystal electron diffraction (MicroED) structure of the MyD88^TIR^ assembly has previously been solved, revealing a two-stranded higher-order assembly of TIR domains. In this work, it is demonstrated that the TIR domain of TLR2, which is reported to signal as a heterodimer with either TLR1 or TLR6, induces the formation of crystalline higher-order assemblies of MyD88^TIR^*in vitro*, whereas TLR1^TIR^ and TLR6^TIR^ do not. Using an improved data-collection protocol, the MicroED structure of TLR2^TIR^-induced MyD88^TIR^ microcrystals was determined at a higher resolution (2.85 Å) and with higher completeness (89%) compared with the previous structure of the MAL^TIR^-induced MyD88^TIR^ assembly. Both assemblies exhibit conformational differences in several areas that are important for signaling (for example the BB loop and CD loop) compared with their monomeric structures. These data suggest that TLR2^TIR^ and MAL^TIR^ interact with MyD88 in an analogous manner during signaling, nucleating MyD88^TIR^ assemblies uni­directionally.

## Abstract 2

This study addresses the structural basis of TIR-domain-mediated signal transduction in Toll-like receptor 2 pathways. It is demonstrated that TLR2, but not TLR1 or TLR6, TIR domains induce the formation of MyD88 TIR-domain assemblies *in vitro*. The MicroED structure of TLR2-induced MyD88 TIR assemblies determined at 2.85 Å resolution highlights conformational changes that are crucial for signaling and provides evidence that TLR2 can directly interact with MyD88 to initiate TLR signaling.

## 1. Introduction

The immune system induces host defenses against microbial diseases. It consists of two components: innate immunity and acquired immunity. Both components assist the body in recognizing non-self microbes and activating immune responses to eliminate the invading organism (Takeda & Akira, 2005 ▸; Akira *et al.*, 2006 ▸). The innate immune system is the primitive form of host defense and is present in most multicellular organisms (Medzhitov & Janeway, 2000 ▸). Toll-like receptors (TLRs) are pattern-recognition receptors (PRRs) that identify endogenous danger-associated molecular patterns (DAMPs) generated by dying or injured cells and evolutionarily conserved pathogen-associated molecular patterns (PAMPs) from invading pathogens (Dinarello, 2011 ▸; Kawai & Akira, 2010 ▸). TLRs are transmembrane proteins that comprise three distinct protein domains: an external leucine-rich-repeat (LRR) domain, a transmembrane (TM) domain and an intracellular Toll/interleukin-1 receptor (TIR) domain (Jiménez-Dalmaroni *et al.*, 2016 ▸; Bell *et al.*, 2003 ▸). Recognition of DAMPs and PAMPs by the LRR domains results in TLR dimerization, bringing together the intracellular TIR domains. This complex subsequently recruits intracellular TIR domain-containing adaptor proteins such as MAL (MyD88 adaptor-like protein) and MyD88 (myeloid differentiation primary response gene 88) through specific interactions between TIR domains, initiating downstream signaling (Nimma *et al.*, 2021 ▸). Recent studies on MAL TIR domains (MAL^TIR^) demonstrate that MAL^TIR^ self-assembles into filaments and nucleates the assembly of MyD88 TIR domains (MyD88^TIR^) into crystalline arrays (Clabbers *et al.*, 2021 ▸; Ve *et al.*, 2017 ▸). The formation of these higher-order assemblies, also called ‘signalosomes’ or ‘supramolecular organizing centers’, results in a mechanism termed ‘signaling by cooperative assembly formation’ (SCAF), in which receptor oligomerization leads to the recruitment and oligomerization of downstream adaptor proteins and effector enzymes to form large protein complexes (Hauenstein *et al.*, 2015 ▸; Kagan *et al.*, 2014 ▸; Nanson *et al.*, 2019 ▸; Nimma *et al.*, 2021 ▸; Wu, 2013 ▸; Yin *et al.*, 2015 ▸).

Both MAL and MyD88 TIR-domain higher-order structures are composed of ‘proto-filaments’ that consist of two parallel strands of TIR-domain subunits arranged in a head-to-tail fashion. This arrangement is largely mediated by the intrastrand BE (BB-loop and αE) and interstrand BCD (αB, αC and αD) interfaces. The interactions highlight a signal-amplification mechanism in TLR signaling pathways in which the TLR, MAL and MyD88 TIR domains undergo a sequential and cooperative assembly process to form a higher-order TIR-domain signalosome. This assembly initiates formation of the downstream complex termed the ‘myddosome’, which consists of the death domains (DDs) of MyD88 and the kinases IRAK2 and IRAK4, leading to proximity-dependent activation of these kinases (Clabbers *et al.*, 2021 ▸; Ve *et al.*, 2017 ▸).

TLR2 forms functional heterodimers with TLR1 and TLR6, which recognize a variety of lipids and cell-wall components, with TLR2/1 and TLR2/6 displaying a preference for microbial triacylated or diacylated lipopeptides, respectively (Oliveira-Nascimento *et al.*, 2012 ▸). Activating these signaling pathways is crucial for the clearance of pathogens and the induction of the adaptive immune response (Takeda & Akira, 2005 ▸; Akira *et al.*, 2006 ▸; Botos *et al.*, 2011 ▸; Kawasaki & Kawai, 2014 ▸). Over the past two decades, research has shown that TLR2 also mediates the pathogenesis of liver diseases such as non-alcoholic steatohepatitis and alcoholic liver disease (Kiziltas, 2016 ▸). Increased expression of TLR2 has also been found in microglia surrounding amyloid β (Aβ) plaques in brains of human Alzheimer’s disease (AD) patients and AD mouse models. Aβ cannot trigger an inflammatory response in TLR2-deficient mice, suggesting that TLR2 plays a significant role in some forms of AD (Jana *et al.*, 2008 ▸; Letiembre *et al.*, 2009 ▸).

Most detailed three-dimensional structural insights into TIR domains have been obtained through single-crystal X-ray diffraction analyses (Nimma *et al.*, 2021 ▸). However, X-ray crystallography relies on large, well ordered crystals (Smyth & Martin, 2000 ▸), which are challenging to produce for certain types of biological samples (Fromme & Spence, 2011 ▸). Microcrystal electron diffraction (MicroED) allows diffraction data collection from submicrometre-sized three-dimensional crystals. This technique, which involves collecting diffraction data from crystals using a low-dose electron beam in a cryo-transmission electron microscope, has been widely applied across various samples, including peptide and protein crystals (Clabbers *et al.*, 2022 ▸; Danelius *et al.*, 2021 ▸; Clabbers & Xu, 2021 ▸; Huang *et al.*, 2021 ▸; Xu *et al.*, 2019 ▸; Gemmi *et al.*, 2019 ▸; Liu *et al.*, 2017 ▸). One of the first novel protein structures solved by MicroED corresponded to the MAL^TIR^-induced MyD88^TIR^ higher-order assembly (Clabbers *et al.*, 2021 ▸). The structure was determined at a resolution of 3.0 Å with an overall completeness of 73.7% owing to the preferred orientation of the flat, plate-like crystals on the electron microscopy (EM) grid. When crystals exhibit preferred orientation, it is difficult to sample the entire reciprocal lattice of the crystal by repeating MicroED data collection over the same rotation range on different crystals. For these kinds of samples, optimization of data-collection procedures is necessary to increase data completeness.

Here, we set out to structurally characterize the TLR2^TIR^-induced MyD88^TIR^ higher-order assembly using MicroED. We found that MyD88^TIR^ microcrystals are induced by the TIR domain of TLR2, but not its binding partners TLR1 or TLR6. The structure is highly similar to the MAL^TIR^-induced MyD88^TIR^ higher-order assembly (Clabbers *et al.*, 2021 ▸). Using a more detailed MicroED data collection, involving a systematic collection of small wedges of data across a larger rotation range, we were able to determine the crystal structure with higher data completeness (89.2%) than the structure reported previously. In addition, collecting data on a microscope operating at a higher accelerating voltage (300 kV compared with 200 kV) led to reduced radiation damage for the same total fluence (Peet *et al.*, 2019 ▸). This expanded the rotation range for which high-resolution spots could be collected within a single data set and enabled a higher overall resolution (2.85 Å) for the final merged data set compared with the structure reported previously. This structure highlights conformational changes in critical regions responsible for MyD88^TIR^ assembly. The findings provide valuable insights into the structural basis of TLR-mediated immune responses, which will facilitate the development of new strategies to combat immunity-related disorders.

## 2. Materials and methods

### 2.1. Expression and purification of TLR1^TIR^, TLR2^TIR^, TLR6^TIR^, MyD88^TIR^, MAL^TIR^ and MyD88^TIR_ΔHIS^

MyD88^TIR_ΔHIS^ was generated by inserting a TEV (Tobacco etch virus) protease cleavage site (ENLYFQSAG) into the previously described MyD88^TIR^ construct (residues 155–296 in pET-28b, C-terminal 6×His tag; Ve *et al.*, 2017 ▸) using a Q5 Site-Directed Mutagenesis Kit (NEB). Auto-induction media (Studier, 2005 ▸) containing either 50 µg ml^−1^ kanamycin or 100 µg ml^−1^ ampicillin were utilized to grow *Escherichia coli* BL21 (DE3) cells expressing MyD88^TIR^ (Ve *et al.*, 2017 ▸), MAL^TIR^ (residues 79–221 in pMCSG7, N-terminal 6×His tag and c-Myc tag; Ve *et al.*, 2017 ▸), TLR2^TIR^ (residues 629–784 in pMCSG7, N-terminal 6×His tag), TLR1^TIR^ (residues 625–786 in pMCSG7, N-terminal 6×His tag), TLR6^TIR^ (residues 637–783 in pMCSG7, N-terminal 6×His tag) and MyD88^TIR_ΔHIS^. The cells were cultured at 30–37°C until they entered the mid-exponential phase (OD_600_ of 0.6–0.8). The cultures were subsequently grown for approximately 16 h at 15–20°C prior to harvesting. The cells were lysed in 50 m*M* HEPES pH 7–8, 500 m*M* NaCl, 1 m*M* dithiothreitol (DTT) via sonication. The samples were clarified by centrifugation for 30 min at ∼27 000*g*. The soluble lysate was loaded onto a 5 ml HisTrap FF column (Cytiva). After sample loading, the column was washed with 15 column volumes (CV) of buffer consisting of 50 m*M* HEPES pH 7–8, 500 m*M* NaCl, 1 m*M* DTT, 30 m*M* imidazole. The bound protein was then eluted using a linear gradient of imidazole ranging from 30 to 250 m*M*. An additional TEV protease cleavage step before size-exclusion chromatography (SEC) was conducted after the expression and purification of MyD88^TIR_ΔHIS^. After IMAC, target protein-containing fractions were combined into SnakeSkin dialysis tubing with 10K molecular-weight cutoff (Thermo Fisher Scientific) and dialyzed against 3 l of gel-filtration (GF) buffer (10 m*M* HEPES pH 7.5, 150 m*M* NaCl, 1 m*M* DTT) at 4°C for 60–120 min to dilute the high concentration of imidazole from the elution buffer. TEV protease was added to the tubing at a molar ratio of 1:20 (TEV protease:target protein) and dialyzed overnight to cleave the 6×His tag. After the overnight dialysis, the solution containing the target protein was loaded onto a 5 ml HisTrap FF column to remove contaminant proteins, uncleaved target proteins and excess TEV protease. The flowthrough was concentrated and loaded onto a HiLoad 26/600 Superdex 75 pg column (Cytiva) pre-equilibrated with GF buffer. Peak fractions were pooled and concentrated to a final concentration of 2–10 mg ml^−1^ using a 10K molecular-weight cutoff Amicon ultracentrifugal filter (Merck Millipore). The purified protein was then flash-frozen in liquid nitrogen and kept at −80°C for future use.

### 2.2. Crystallization of MyD88^TIR^

TLR2^TIR^-induced MyD88^TIR^ microcrystals were prepared by incubating TLR2^TIR^ (6–120 µ*M*) with MyD88^TIR^ (60 µ*M*) in GF buffer at 30°C for 60–120 min. MAL^TIR^-induced MyD88^TIR^ microcrystals were prepared by incubating MAL^TIR^ (6 µ*M*) with MyD88^TIR^ (60 µ*M*) in GF buffer at 30°C for 60–120 min.

### 2.3. Turbidity-based polymerization assay

In a UV-Star microplate (Greiner Bio-One), samples of protein mixtures were prepared in GF buffer to a final volume of 100 µl; GF buffer was used as an assay blank. The plate was placed in a SpectraMax 250 microplate reader (Molecular Devices) and incubated at 30°C for 1–2 h. Each sample was prepared in duplicate, and the absorbance was measured at 350 nm every 30 s for 1 h. The samples were immediately transferred to EM grids and visualized using negative-stain EM.

### 2.4. MicroED sample preparation and data collection

Vitrified samples for MicroED data collection were prepared by depositing 3 µl of microcrystal solution comprising 30 µ*M* TLR2^TIR^ and 60 µ*M* MyD88^TIR^ onto a Quantifoil 1.2/1.3 (300 mesh) Cu holey carbon EM grid that had been previously glow-discharged for 40 s at 20 mA using a PELCO easiGlow. 90 µ*M* TLR2^TIR^ and 60 µ*M* MyD88^TIR^ were mixed and incubated at 30°C. MicroED samples were prepared by freezing the mixed solution after a series of different incubation times in steps of 5 min from 30 min to 1 h after mixing. Prior to deposition, the sample was homogenized in the mother liquor by gently pipetting the mixture up and down. Excess liquid was removed by double-sided blotting for 3 s. The grid was then immediately vitrified in liquid ethane using an FEI Vitrobot Mark IV (ThermoFisher Scientific) operating at blot force 3 and blot time 3 s at 4°C and 80% humidity. MicroED data were collected using a Titan Krios cryo-transmission electron microscope (TEM; ThermoFisher Scientific) operating at 300 kV and equipped with a Ceta-D CMOS detector. Screening and MicroED data collection, using the continuous-rotation method (Nannenga *et al.*, 2014 ▸), were performed using *EPU-D* (ThermoFisher Scientific). Diffraction data were collected using a parallel beam of ∼1 µm in size with a 50 µm C2 aperture and spot size 10. The oscillation step was controlled at a fixed value between 0.5° and 1.0°. The dose per frame was 0.138 e Å^−2^ with 1 s exposure time, giving an average total exposure per data set of 5.6 e Å^−2^. Due to preferred orientation of the flat, plate-like crystals, multiple MicroED data sets were collected to cover an overall rotation range of −65° to 65°. The data sets were systematically collected across this rotation range in wedges of 40° on average. Each data set was collected at a rotation range that ensured overlap with the previous and the subsequent wedge. This overlap enabled calculation of correlation coefficients between the data sets during the merging process.

### 2.5. MicroED data processing and structure determination

The diffraction data from 20 crystals were processed using *X-ray Detector Software* (*XDS*) and *X-ray Scaling Program* (*XSCALE*) (Kabsch, 2010 ▸) to obtain integrated and scaled data, which were subsequently merged using *AIMLESS* (Evans, 2006 ▸). The resolution cutoff was chosen based on an average *I*/σ(*I*) ratio of >1.0 and a CC_1/2_ of >0.30. The corresponding data statistics are provided in Table 1 ▸. The crystal structure of MyD88^TIR^ was solved by molecular replacement using *Phaser* (McCoy *et al.*, 2007 ▸) in the *Phenix* software suite. The previously solved crystal structure of MAL^TIR^-induced MyD88^TIR^ by MicroED (Clabbers *et al.*, 2021 ▸; PDB entry 7beq) was used as the search model. The model of the TLR2^TIR^-induced MyD88^TIR^ microcrystal was iteratively built and refined using *Coot* (Emsley *et al.*, 2010 ▸) and *phenix.refine* (Afonine *et al.*, 2012 ▸). The refinement process was performed using a test set of ∼5% of the reflections to calculate *R*_free_. The refinement strategy used electron scattering factors, group *B*-factor and translation/libration/screw (TLS) parameter refinement, Ramachandran restraints, optimization of data versus stereochemistry, and atomic displacement parameter weighting. The structure was validated using *MolProbity* (Williams *et al.*, 2018 ▸) in the *Phenix* software suite. The statistics of the final refined model are shown in Table 2 ▸. No reflections were filled in for map calculation. All selected data sets have been deposited with Zenodo and are available at https://doi.org/10.5281/zenodo.10722078.

### 2.6. Structure analyses

Structural analyses, alignments, root-mean-square deviation (r.m.s.d.) calculations and figure preparation were carried out using *PyMOL* (version 2.2.3; Schrödinger).

### 2.7. Nano-gold labeling assay and negative-stain EM

Mixtures of TLR2^TIR^ and MyD88^TIR^ were loaded onto an EM grid and incubated for 2 min at room temperature. The grid was subsequently washed with GF buffer for 10 s, placed onto a droplet of 5 nm Ni-NTA-Nanogold label (Nanoprobes, USA) and incubated for 30 min at room temperature. The grid was then washed with 50 m*M* HEPES pH 7.5, 150 m*M* NaCl containing different concentrations of imidazole (8, 20 and 30 m*M*; 1 min incubation for each), followed by water rinsing. Grids were stained with 1% uranyl acetate for 1 min. During each step, excess liquid was removed using filter paper. Samples were visualized using a Jeol JEM-1011 or Hitachi HT 7700 TEM at an accelerating voltage of 80–120 kV.

### 2.8. Crystal-growth assay using time-lapse imaging

TLR2^TIR^-induced MyD88^TIR^ microcrystal seeds were produced by incubating TLR2^TIR^ with MyD88^TIR^ (60:60 µ*M*) at 30°C for 20 min. The seeds were centrifuged for 5 min at 2000*g* and then rinsed three times with 250 µl GF buffer. Seeds were resuspended in 100 µl GF buffer and were then diluted 1:3200 in GF buffer. 5 µl of the diluted seeds was transferred into each well of a 96-well imaging plate (ibiTreat sterile, Ibidi) containing 45 µl 60 µ*M* MyD88^TIR^. The plate was centrifuged at 1500*g* for 5 min before being transferred to the microscope for imaging. The plate was incubated at 30°C during imaging. Imaging was performed using a Nikon Eclipse Ti2 inverted microscope. Differential interference contrast (DIC) images were captured with a 40× objective lens using 1.5× magnification.

## 3. Results

### 3.1. TLR2^TIR^, and not TLR1^TIR^ or TLR6^TIR^, induces the formation of crystalline higher-order assemblies of MyD88^TIR^

**Figure 1:**
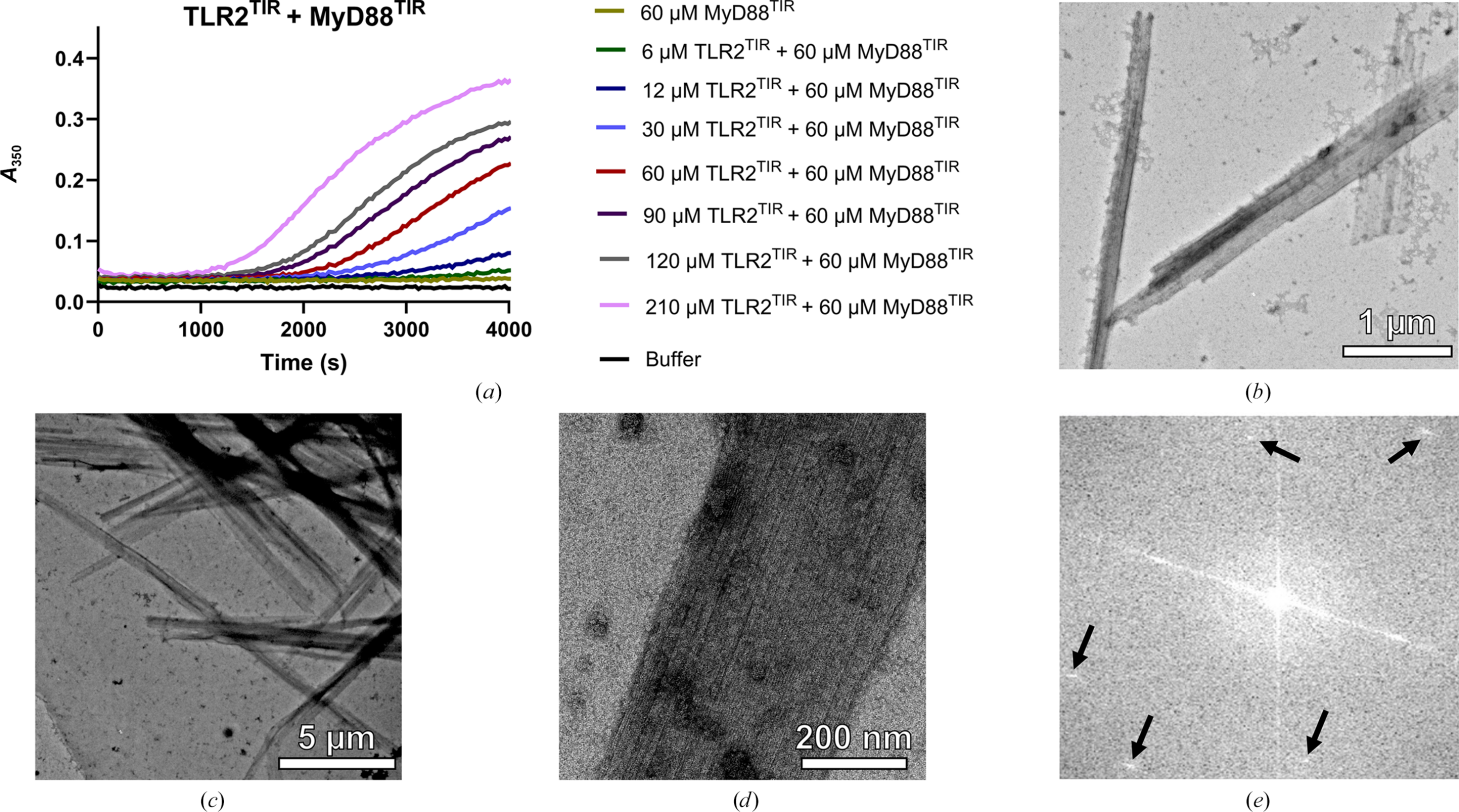
Incubation of TLR2^TIR^ with MyD88^TIR^ induces higher-order assembly formation. (*a*) Turbidity-based polymerization assays testing the effect of increasing concentrations of TLR2^TIR^ on higher-order assembly formation. (*b*–*e*) Negative-stain EM analysis reveals higher-order assemblies with ordered crystalline arrays. (*b*) 30 µ*M* TLR2^TIR^ mixed with 60 µ*M* MyD88^TIR^. (*c*) 120 µ*M* TLR2^TIR^ mixed with 60 µ*M* MyD88^TIR^. (*d*) 60 µ*M* TLR2^TIR^ mixed with 60 µ*M* MyD88^TIR^ [shown at higher magnification than (*b*) and (*c*)]. (*e*) FFT of images containing assemblies revealed the assemblies possess a crystalline lattice, as shown by the presence of reflections (indicated by black arrows). Assays were repeated at least twice with similar results.

**Figure 2:**
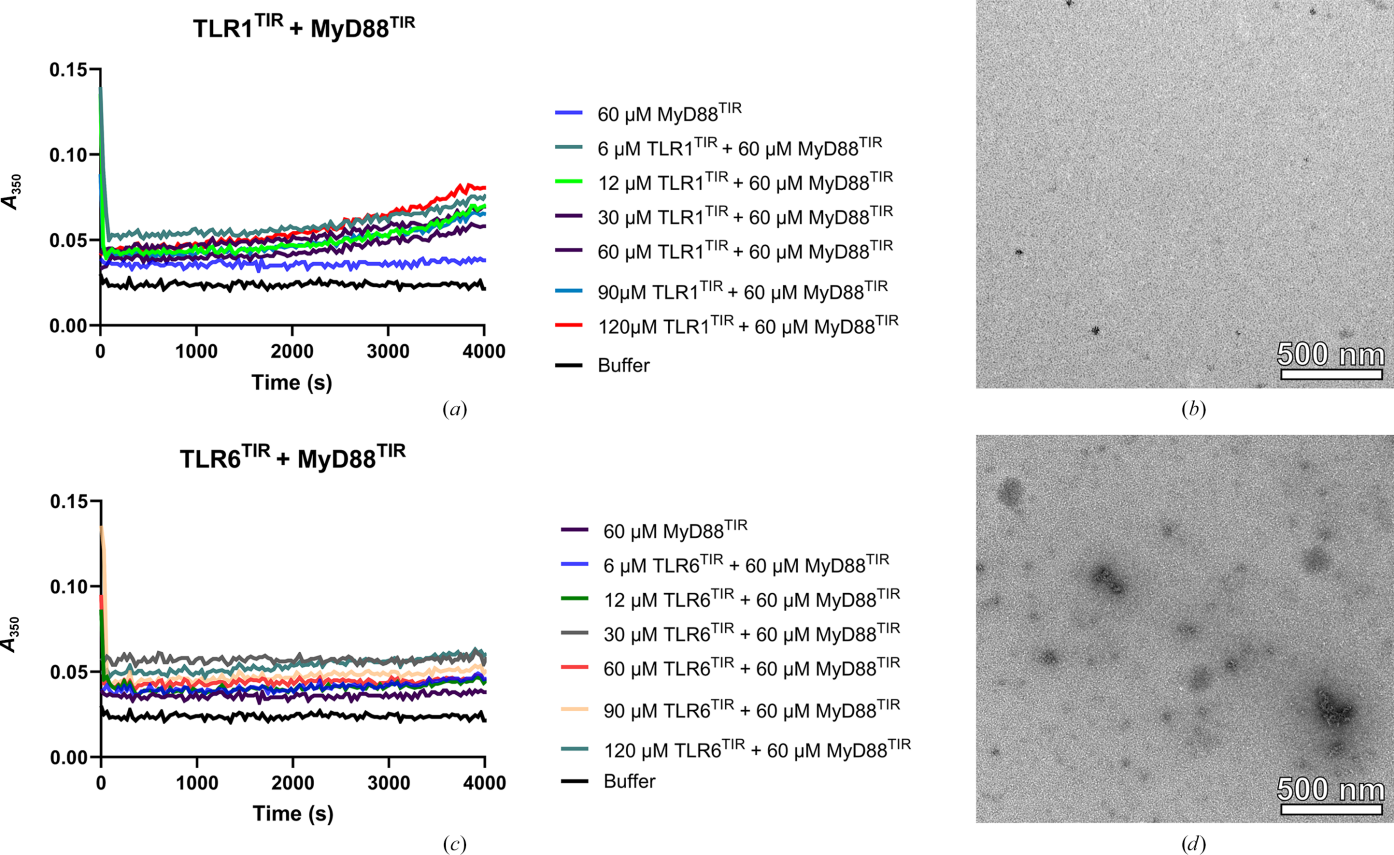
Incubation of TLR1^TIR^ or TLR6^TIR^ with MyD88^TIR^ does not induce higher-order assembly formation. No significant increase in turbidity was observed and negative-stain EM did not show the presence of higher-order assemblies. (*a*) Turbidity-based polymerization assays testing the effect of TLR1^TIR^ on higher-order assembly formation. (*b*) Negative-stain EM analysis of 30 µ*M* TLR1^TIR^ mixed with 60 µ*M* MyD88^TIR^. (*c*) Turbidity-based polymerization assays testing the effect of TLR6^TIR^ on MyD88^TIR^ higher-order assembly formation. (*d*) Negative-stain EM of 30 µ*M* TLR6^TIR^ mixed with 60 µ*M* MyD88^TIR^. Assays were repeated at least twice with similar results.

We used a turbidity-based polymerization assay to detect the formation of insoluble higher-order assemblies (Ve *et al.*, 2017 ▸) between TIR domains of receptors (TLR1, TLR2 and TLR6) and the adaptor MyD88. We observed that the solution became cloudy when MyD88^TIR^ was mixed with TLR2^TIR^, indicating the presence of assemblies. With increasing amounts of TLR2^TIR^, polymerization appeared to proceed more quickly, indicating a concentration-dependent mechanism of assembly formation (Fig. 1 ▸*a*). Negative-stain EM revealed the presence of crystalline arrays (microcrystals or nano­crystals; Figs. 1 ▸*b*–1 ▸*d*). Fast Fourier transform (FFT) of the TEM images confirmed the presence of microcrystals (Fig. 1 ▸*e*). Turbidity assays showed that these microcrystals formed at a concentration of TLR2^TIR^ that was approximately eightfold higher than the concentration of MAL^TIR^ used previously to induce MyD88^TIR^ microcrystals (Ve *et al.*, 2017 ▸), thus indicating that MyD88 preferentially interacts with MAL^TIR^. When incubating MyD88^TIR^ with either TLR1^TIR^ or TLR6 ^TIR^, the formation of microcrystals was not observed as the solution remained clear (Figs. 2 ▸*a* and 2 ▸*c*). This was further supported by negative-stain EM, which did not show the presence of higher-order assemblies (Figs. 2 ▸*b* and 2 ▸*d*). These findings imply that the TIR domains of TLR1 and TLR6 do not induce MyD88^TIR^ higher-order assembly formation and are therefore unlikely to interact directly with MyD88^TIR^.

### 3.2. TLR2^TIR^ nucleates MyD88^TIR^ assembly formation unidirectionally

#### 3.2.1. Gold labeling cannot distinguish whether TLR2^TIR^ molecules initiate crystallization or are incorporated throughout TLR2^TIR^-induced MyD88^TIR^ microcrystals

**Figure 3:**
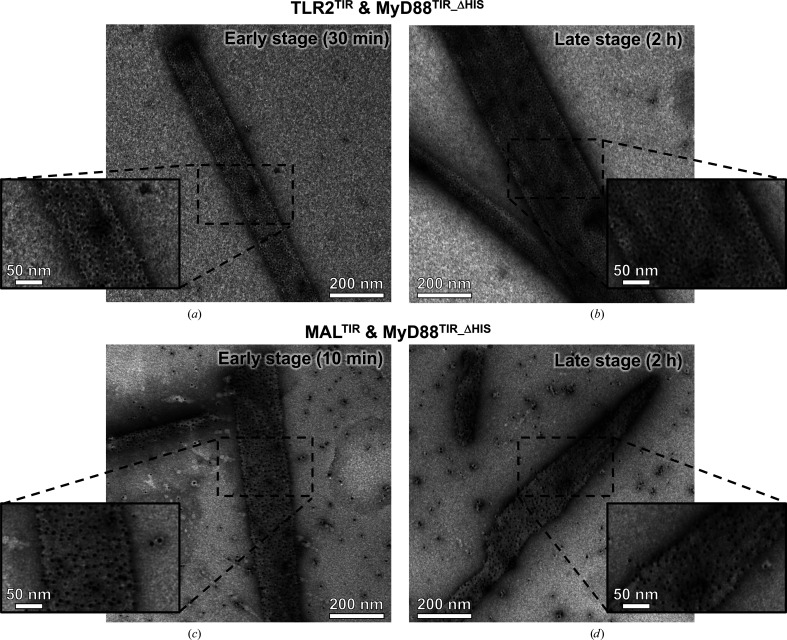
Nano-gold labeling analysis of TLR2^TIR^-induced and MAL^TIR^-induced MyD88^TIR_ΔHIS^ microcrystals. Gold-labeled domains are found to cover the microcrystals at both early (*a*, *c*) and late (*b*, *d*) stages of crystal formation.

To investigate the formation of microcrystals, a gold-labeling experiment was conducted on TLR2^TIR^ containing a His tag (TLR2^TIR^) and MyD88^TIR^ without a His tag (MyD88^TIR_ΔHIS^) using Ni-NTA-Nanogold, which specifically labels His-tagged proteins. We incubated 90 µ*M* TLR2^TIR^ with 60 µ*M* MyD88^TIR_ΔHis^ on a TEM grid at an early (30 min) and a late (2 h) crystallization time point. Gold-labeled TLR2^TIR^-induced microcrystals were observed at both early and late stages of crystal formation (Figs. 3 ▸*a* and 3 ▸*b*), indicating that TLR2^TIR^ may either be incorporated throughout the microcrystals or only located on the surface of the microcrystals. We then sought to compare these results with MAL^TIR^-induced MyD88^TIR_ΔHIS^ microcrystals. A gold-labeling experiment was also conducted on the microcrystals formed by incubating 6 µ*M* His-tagged MAL^TIR^ with 60 µ*M* MyD88^TIR_ΔHIS^. Similarly, gold-labeled MAL^TIR^ microcrystals were observed at both early (10 min) and late (2 h) stages during crystal formation (Figs. 3 ▸*c* and 3 ▸*d*). Previous research (Clabbers *et al.*, 2021 ▸) suggested that MAL^TIR^ only initiates the formation of MyD88^TIR^ microcrystals and that MAL^TIR^ molecules do not incorporate into the crystals. We conclude that under the conditions used gold labeling cannot distinguish whether TLR2^TIR^ and MAL^TIR^ molecules are incorporated into the MyD88^TIR_ΔHIS^ microcrystals or merely initiate MyD88^TIR_ΔHIS^ microcrystal formation, and the labeling of microcrystals may occur due to TLR2^TIR^ or MAL^TIR^ coating the surface of the microcrystals or a nonspecific interaction of Ni-NTA-Nanogold with proteins lacking a His tag.

#### 3.2.2. A time-resolved study of TLR2^TIR^-induced MyD88^TIR^ microcrystals suggests that TLR2^TIR^ does not co-polymerize with MyD88^TIR^ in microcrystals

**Figure 4:**
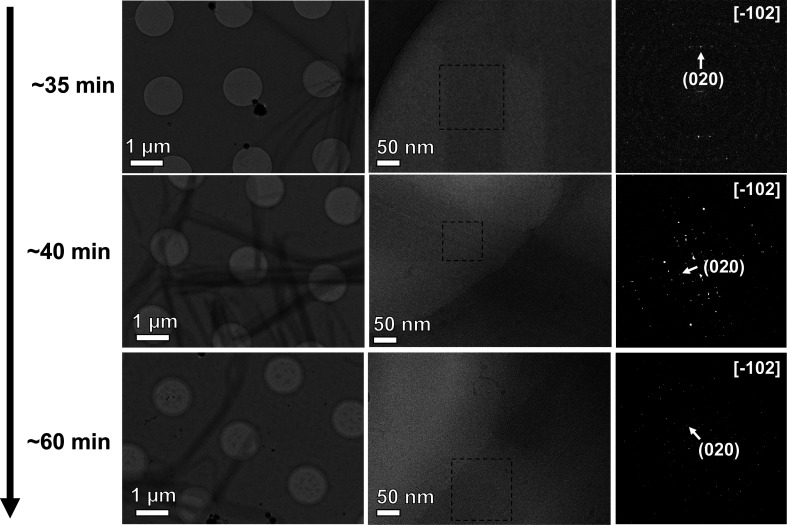
TLR2^TIR^-induced MyD88^TIR^ microcrystals at different time points. Small clusters of crystals formed at the early stage of crystallization. After ∼35 min of incubation, ultrathin, weakly crystalline ribbon-like crystals formed, as revealed by the calculated FFT. By ∼40 min, the ribbons grew in thickness and became fully crystalline. Analysis of the calculated FFT indicated the fully grown crystals were oriented along the [−102] zone axis, with consistent *d*-spacing of the (020) plane throughout crystal growth. The boxed area indicates the portion of the microcrystal used for FFT.

To further study TLR2^TIR^-induced MyD88^TIR^ microcrystal formation, a time-resolved study was performed to investigate the microcrystal-formation process (Fig. 4 ▸). MicroED samples (90 µ*M* TLR2^TIR^ and 60 µ*M* MyD88^TIR^) were prepared by freezing the mixed solution at different incubation times in steps of 5 min from 30 min to 1 h after mixing. Small clusters of crystals formed at the early stage of crystallization. After ~35 min incubation, very thin, ribbon-like microcrystals had formed with a similar length to the fully grown microcrystals. Imaging these ribbon-like crystals at higher magnification revealed they were ultrathin, as the contrast was very weak. FFT of the micrographs revealed that these crystals were weakly crystalline, with one clearly visible Fourier peak that could be attributed to (020) crystal planes. These crystal planes were perpendicular to the direction of the missing wedge in the MicroED data, indicating that the orientation is consistent with the preferred orientation displayed by the fully formed crystals (Supplementary Fig. S1). After ∼40 min of incubation the microcrystals had grown in thickness and were fully crystalline, as revealed by Fourier transform (Fig. 4 ▸). Analysis of the FFT patterns of the fully grown crystals determined that the microcrystals were lying along the [−102] zone axis, which was in agreement with the diffraction data collected from TLR2^TIR^-induced MyD88^TIR^ microcrystals for structure determination. Although the initial crystals were only weakly crystalline, the *d*-spacing of the (020) plane was consistent throughout all time points and the diffraction data did not reveal any differences in the crystallographic lattice that would indicate the presence of crystalline TLR2^TIR^ assemblies in any of the small initial crystals or the final MyD88^TIR^ microcrystals. In contrast to our prior investigations with gold labeling, these results suggest that TLR2^TIR^ is only involved in the nucleation of MyD88^TIR^ microcrystals.

#### 3.2.3. Real-time monitoring of TLR2^TIR^-induced MyD88^TIR^ microcrystal formation reveals unidirectional assembly formation

**Figure 5:**
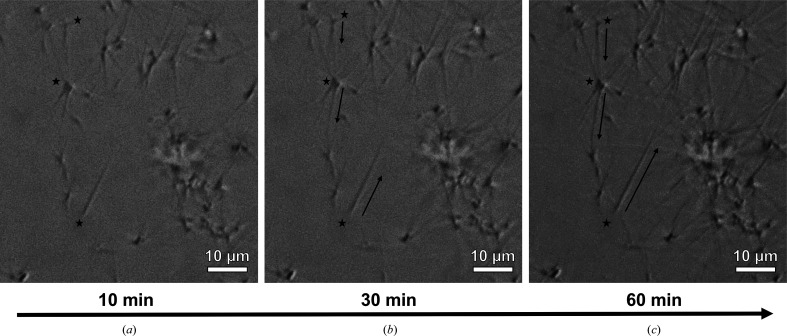
Time-lapse imaging of MyD88^TIR^ microcrystal formation nucleated by TLR2^TIR^. Seeds were rinsed to remove free TLR2^TIR^ and then incubated with MyD88^TIR^. DIC images were taken at different time points: (*a*) 10 min, (*b*) 30 min and (*c*) 60 min. Unidirectional elongation of microcrystals was observed. Selected microcrystals or clusters of microcrystals are indicated by a black star; black arrows indicate the direction of crystal elongation.

A crystal-growth experiment using differential interference contrast (DIC) was also conducted to capture TLR2^TIR^-induced MyD88^TIR^ higher-order assembly formation. Previous work revealed that the MAL^TIR^-induced MyD88^TIR^ higher-order assembly was initiated by His-tagged MAL^TIR^ molecules acting as nucleants (Clabbers *et al.*, 2021 ▸). Short MAL^TIR^-induced MyD88^TIR^ crystal seeds were washed multiple times to eliminate excess MAL^TIR^ and were then incubated with additional MyD88^TIR^. After removing excess MAL^TIR^, the MyD88^TIR^ microcrystals kept growing unidirectionally, indicating that MAL^TIR^ is necessary for MyD88^TIR^ assembly nucleation but not for elongation (Clabbers *et al.*, 2021 ▸). To confirm whether a similar nucleation mechanism operates for TLR2^TIR^-induced MyD88^TIR^ assemblies, His-tagged TLR2^TIR^ was used to generate microcrystal seeds. MyD88^TIR^ crystals seeded by His-tagged TLR2^TIR^ displayed elongation from one end only (Fig. 5 ▸). Consistent with our cryo-EM study, small clusters of crystals were also observed due to aggregation of the MyD88^TIR^ seeds. Our results indicate that the receptor (TLR2^TIR^) and the adaptor (MAL^TIR^) both induce a comparable unidirectional elongation mechanism during MyD88^TIR^ assembly formation.

### 3.3. MicroED data acquisition

**Figure 6:**
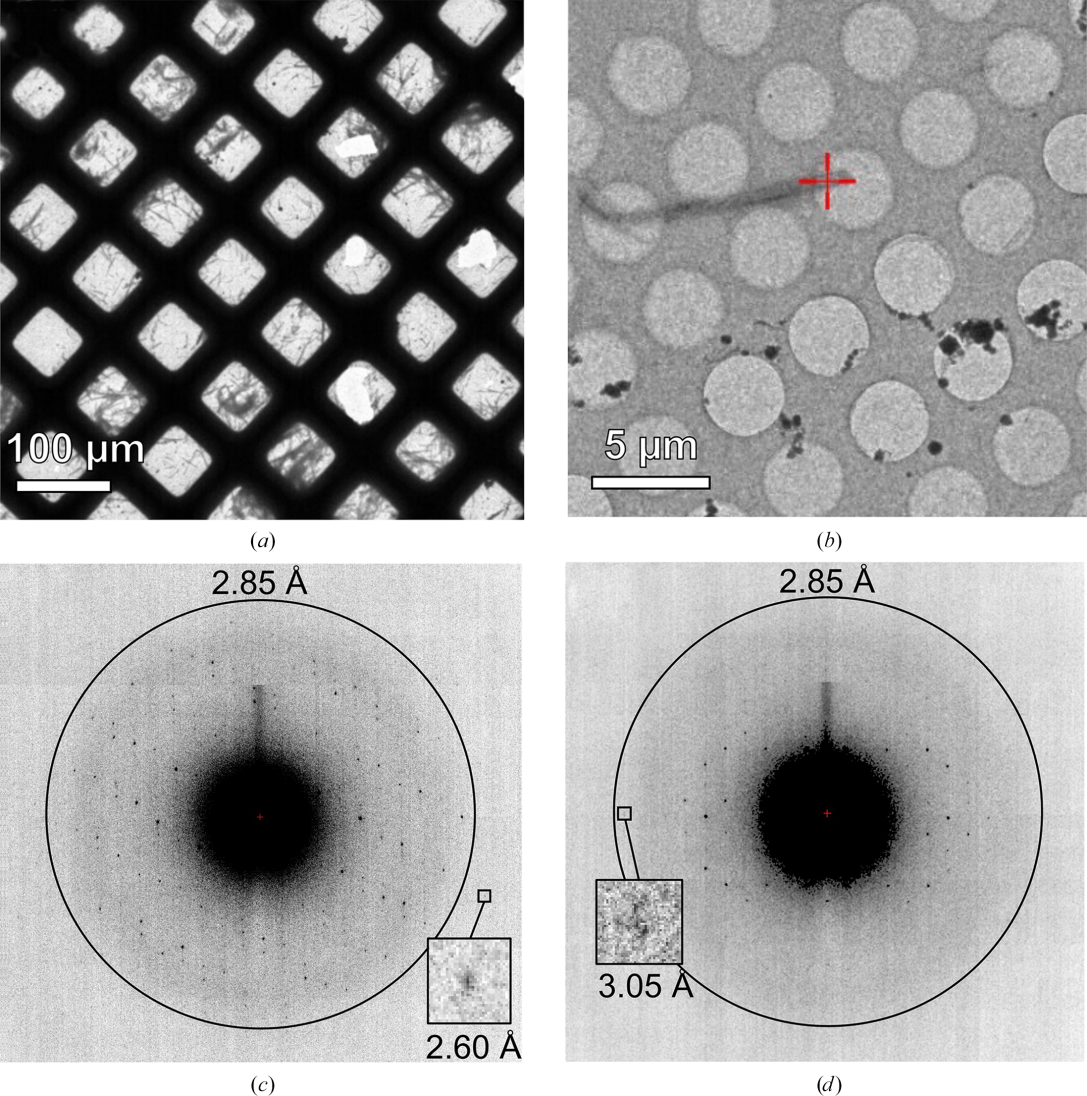
MicroED data collection of TLR2^TIR^-induced MyD88^TIR^ microcrystals. (*a*) Illustration of a sample grid employed for data acquisition. (*b*) An individual MyD88^TIR^ microcrystal resting on the grid. (*c*) Diffraction pattern derived from a single TLR2^TIR^-induced MyD88^TIR^ microcrystal after exposure to a fluence of ∼0.14 e Å^−2^ (0° rotation), showing visual spots up to 2.60 Å resolution, as highlighted in the figure. (*d*) Diffraction pattern towards the end of data collection (∼4.4 e Å^−2^; 32.5° rotation), with a highlighted reflection visible at 3.05 Å resolution. The ring illustrates the data resolution cutoff for processing and final refinement.

TLR2^TIR^-induced MyD88^TIR^ microcrystals were typically 200–300 nm in diameter, making them suitable for MicroED analysis. TLR2^TIR^-induced MyD88^TIR^ microcrystals were deposited onto Quantifoil EM grids and vitrified. MicroED data collection was carried out on crystal growth after a 60 min incubation (Fig. 6 ▸). Due to a preferred orientation of the microcrystals, to achieve reasonable data completeness MicroED data were typically collected in wedges of ∼40° over a large rotation range of the goniometer (−65° to 65°). Loss of high-resolution reflections indicating some degree of radiation damage was observed towards the end of data-collection wedges (Figs. 6 ▸*c* and 6 ▸*d*). Data from 20 crystals were integrated, scaled and merged (Table 1 ▸) to obtain a 2.85 Å resolution data set with a completeness of 89.2%.

### 3.4. Comparison of MyD88^TIR^ structures

**Figure 7:**
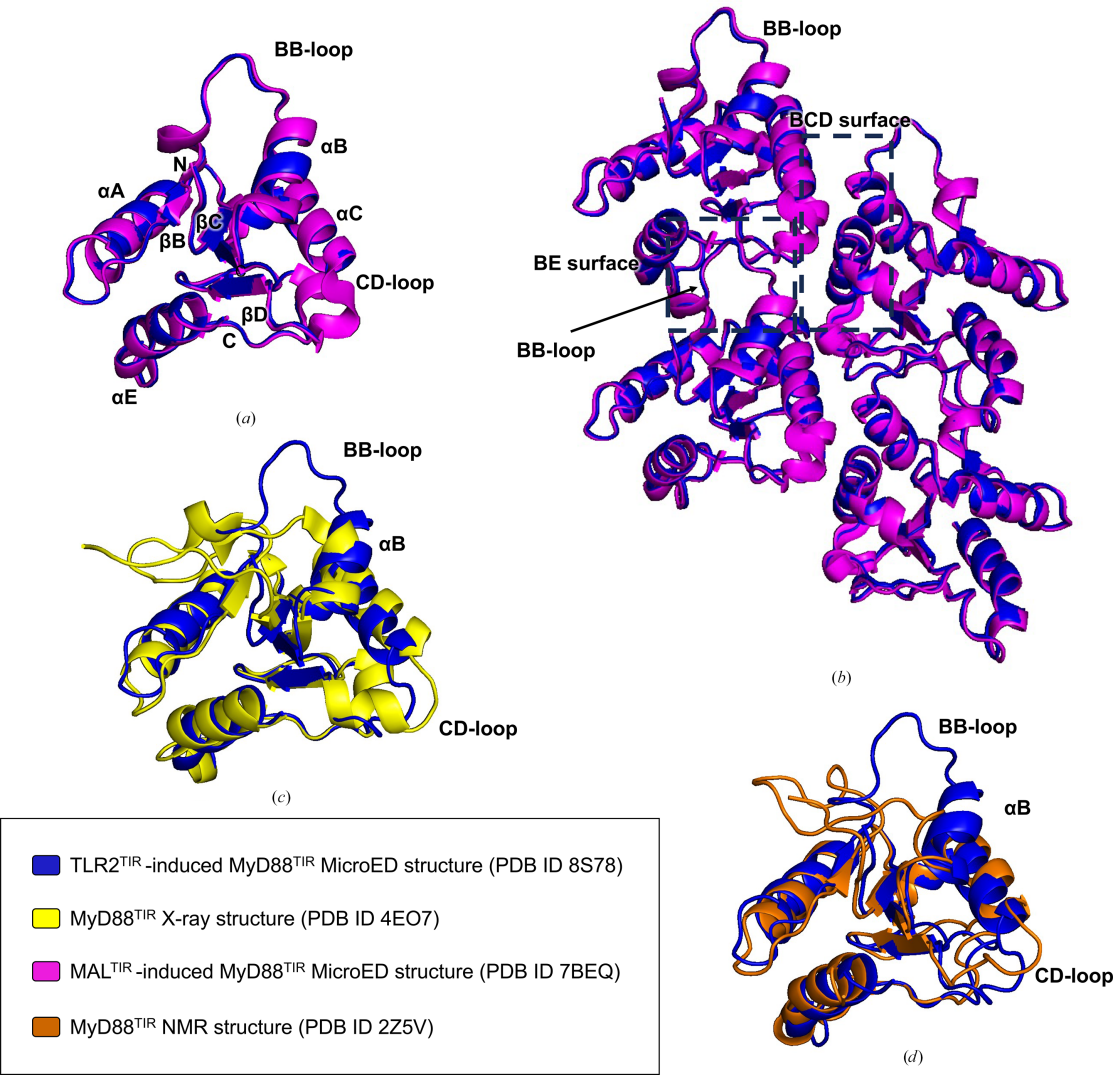
MicroED structure of the TLR2^TIR^-induced MyD88^TIR^ higher-order assembly. (*a*) Superposition of the TLR2^TIR^-induced MyD88^TIR^ (PDB entry 8s78; blue) and the MAL^TIR^-induced MyD88^TIR^ (PDB entry 7beq; magenta) structures. (*b*) Structural alignment of TLR2^TIR^-induced and MAL^TIR^-induced MyD88^TIR^ assemblies (four molecules are shown). (*c*, *d*) Superposition of the TLR2^TIR^-induced MyD88^TIR^ structure (blue) with the MyD88^TIR^X-ray structure (PDB entry 4e07; yellow) (*c*) and MyD88^TIR^ NMR structure (PDB entry 2z5v; orange) (*d*). Significant conformational differences (for example in the BB loop, CD loop and αB helix) are observed in several regions when compared with the X-ray and NMR structures of monomeric proteins.

The structure of TLR2^TIR^-induced MyD88^TIR^ microcrystals was solved by molecular replacement using the previously solved MicroED structure of MAL^TIR^-induced MyD88^TIR^ microcrystals (PDB entry 7beq) as the search model. *Phenix*-generated *MolProbity* statistics of the final refined model are shown in Table 2 ▸. The structures contain only MyD88^TIR^ molecules; the seeding TLR2^TIR^ molecules were not discernible in the electrostatic potential maps. We were able to determine the structure of TLR2^TIR^-induced MyD88^TIR^ microcrystals at a higher resolution (2.85 Å) and with higher completeness (89.2%) compared with the previous MyD88^TIR^ structure induced by MAL^TIR^ (3.00 Å and 73.7%, respectively). Superposition of TLR2^TIR^-induced and MAL^TIR^-induced MyD88^TIR^ microcrystal structures reveals nearly identical structures, with an r.m.s.d. value of 0.35 Å over 129 C^α^ atoms (Fig. 7 ▸*a*; Supplementary Table S1).

The higher-order assembly of TLR2^TIR^-induced MyD88^TIR^ exhibits a crystal-packing pattern comparable to the higher-order assembly of MAL^TIR^-induced MyD88^TIR^, in which two offset parallel strands of MyD88^TIR^ subunits are arranged head to tail. The interfaces between the TIR domains in the strands are mediated through asymmetric interactions. These consist of intrastrand (head-to-tail) interactions occurring between MyD88^TIR^ subunits within each of the strands (referred to as the BE intrastrand interface) and interstrand (lateral) interactions occurring between MyD88^TIR^ subunits of the two strands (referred to as the BCD interstrand interface) (Fig. 7 ▸*b*). Both MyD88^TIR^ structures, induced by either adaptor or receptor TIR domains, reveal conformational differences in several regions (for example the BB loop, CD loop and αB helix) when compared with the X-ray and NMR structures of monomeric MyD88^TIR^ (Figs. 7 ▸*c* and 7 ▸*d*).

## 4. Discussion

Our study provides insights into the molecular mechanisms underlying TLR2-mediated immune responses and the potential involvement of MAL and MyD88 in these pathways. Although MAL is essential for TLR4 responses, previous work has shown that MAL enhances the sensitivity to low ligand concentrations, but is not essential for all responses as MAL-knockout mouse macrophages can respond to TLR2 ligands (Cole *et al.*, 2010 ▸; Kenny *et al.*, 2009 ▸). Cole and coworkers found that TLR2 was able to directly interact with and activate MyD88 signaling in response to phagosomal *Francisella tularensis* independently of MAL. Indeed, the findings of our study indicate that the role of the adaptor protein MAL in TLR2 signaling may not be the same as its role in TLR4 signaling. Our results demonstrate that MyD88^TIR^ can be recruited by TLR2^TIR^, but not by TLR1^TIR^ or TLR6^TIR^, and can form higher-order assemblies without MAL. While not strictly essential for signaling, our data and other available data indicate that MAL may play a role in this assembly through heterodimers (TLR2/1 or TLR2/6) via interaction with TLR1 or TLR6. If recruited, MAL would then facilitate the assembly of MyD88 in conjunction with TLR2.

Structure solution by MicroED revealed that TLR2^TIR^-induced assemblies of MyD88^TIR^ are identical to the MAL^TIR^-induced higher-order assemblies presented in Clabbers *et al.* (2021 ▸). By systematically collecting small wedges of multiple data sets at high flux over a wide range of tilting angles, we improved the data-collection procedure, determining the structure of MyD88^TIR^ assemblies at a higher resolution (2.85 Å) and with greater completeness (∼89%) than the previously reported MicroED structure (Table 1 ▸). Despite observing indications that the employed electron dose (average total exposure of 5.6 e Å^−2^ per data set) resulted in radiation damage during data acquisition, we did not encounter any structural modifications that could have affected accurate interpretation of the electrostatic potential map or the structural model. Glutamate, aspartate and cysteine residues, which can accumulate damage and display site-specific loss of ‘density’ at electron exposures lower than those used in this study (Hattne *et al.*, 2018 ▸), could still be resolved within our 2*mF*_o_ − *DF*_c_ map contoured to 1.0 r.m.s.d. in *Coot* (Emsley *et al.*, 2010 ▸). More sensitive instrumentation allowing a reduced total dose and thus less radiation damage may result in greater data completeness at high resolution and an improved map. While the data completeness of the TLR2^TIR^-induced MyD88^TIR^ structure reported here is greater than that reported for the MAL^TIR^-induced MyD88^TIR^ structure (Table 1 ▸; Clabbers *et al.*, 2021 ▸), data completion is still limited due to a preferred orientation of the microcrystals. A technique to overcome preferred orientation and missing data wedges has recently been reported (Gillman *et al.*, 2023 ▸, 2024 ▸). A combination of this technique and the use of more sensitive instrumentation could be used to reduce the limitations experienced in this, and similar, MicroED studies.

Clabbers *et al.* (2021 ▸) compared the structures of MAL^TIR^ and MyD88^TIR^ monomers (‘signaling-inactive’ forms) and their higher-order complexes (‘signaling-active’ forms). Significant conformational differences were observed in the BB and BC surfaces, notably within the BB loop and αB helix. By contrast, the EE and CD surfaces appeared to be structurally similar. This indicates that the conformational changes occurring in the BB and BC areas are crucial for the activation of TLRs.

Molecular modeling suggests that the EE surface of MyD88^TIR^ monomers preferentially interacts with the BB surface of MAL^TIR^ or another MyD88^TIR^ subunit during the formation of MyD88^TIR^ assemblies, facilitating the nucleation and elongation phases. The interaction triggers a rearrangement of the αB helix and BB loop in the newly added TIR-domain molecule, forming a binding interface for the EE surface of the next MyD88^TIR^ monomer. Introducing new MyD88^TIR^ monomers to an extended assembly through the EE surface only requires minor conformational modifications before binding, indicating a more preferred strategy compared with recruiting through the BB surface, which involves significant structural rearrangements (Clabbers *et al.*, 2021 ▸).

**Figure 8:**
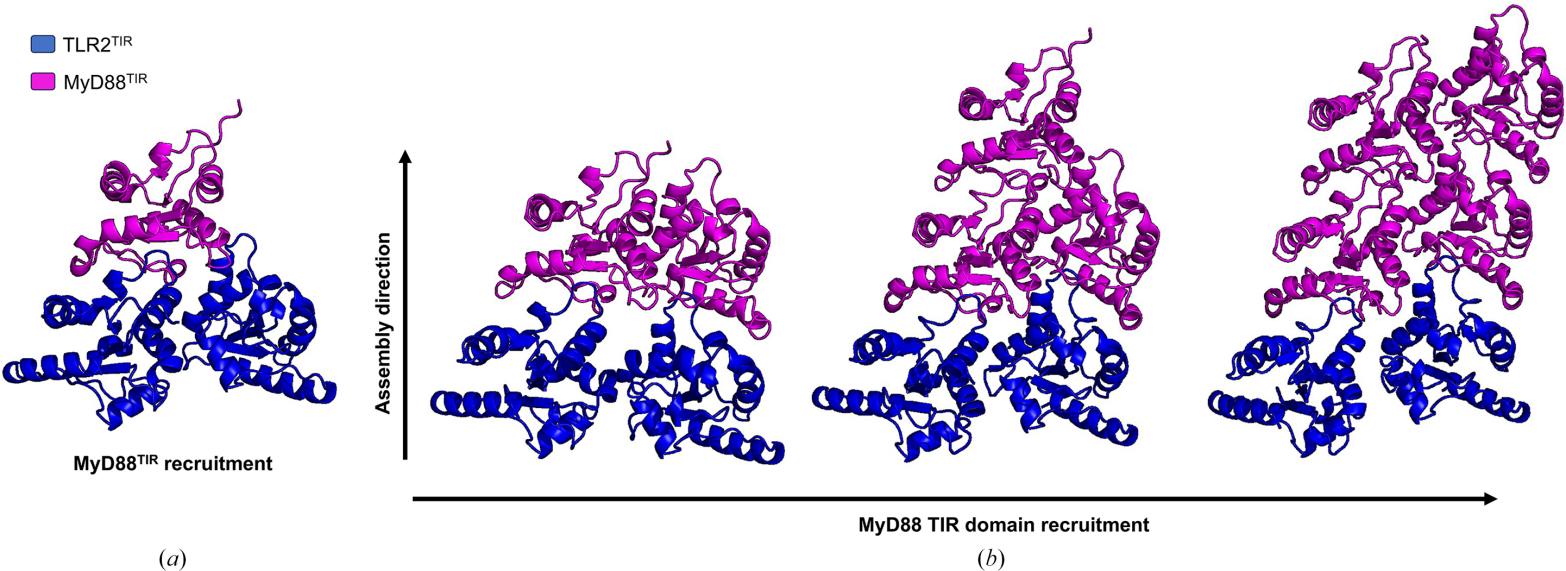
A predicted model of TLR2^TIR^-induced MyD88^TIR^ unidirectional assembly formation. (*a*) Initially, a TLR2^TIR^ homodimer (blue) recruits a single MyD88^TIR^ molecule (magenta). (*b*) This stepwise assembly involves the addition of successive MyD88^TIR^ units (2, 3, 4). The subsequent assembly of MyD88^TIR^ is facilitated by interaction between the EE surface of the incoming monomeric MyD88^TIR^ and the BB surface of the oligomerized MyD88^TIR^ subunit, a process that is vital to both the nucleation and elongation stages.

**Figure 9:**
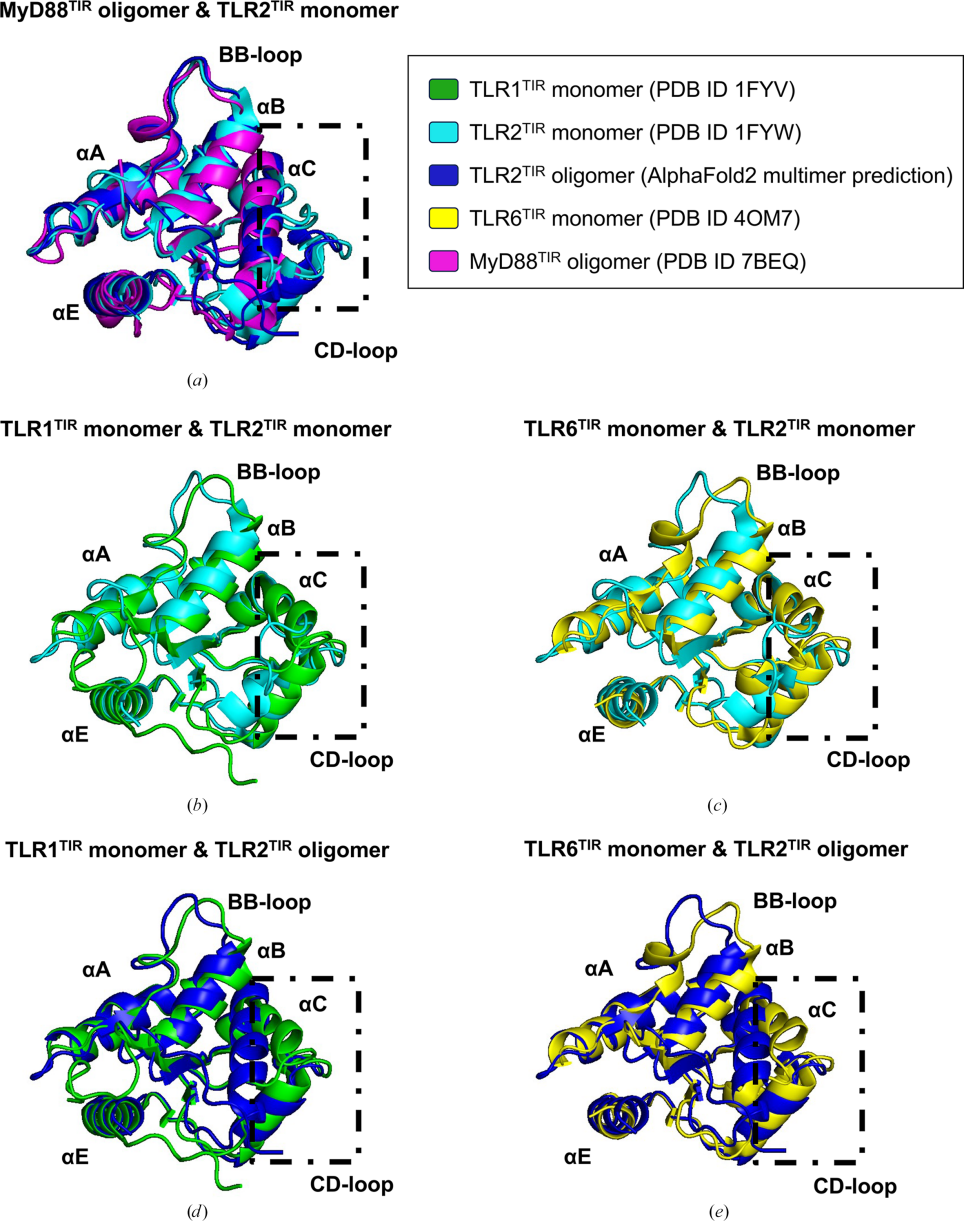
Comparison of MyD88, TLR1, TLR2 and TLR6 TIR-domain structures. (*a*) Superposition of the MAL^TIR^-induced MyD88^TIR^ structure (PDB entry 7beq; magenta) and TLR2^TIR^ from the crystal structure (PDB entry 1fyw; cyan) and *AlphaFold*2-predicted oligomer (blue). (*b*,* c*) Structural alignment of the crystal structures of TLR2^TIR^ and TLR1^TIR^ (PDB entry 1fyv; green) (*b*) and TLR6^TIR^ (PDB entry 4om7; yellow) (*c*). (*d*, *e*) Structural alignment of TLR2^TIR^ from our *AlphaFold*2-predicted oligomer structure and the crystal structures of TLR1^TIR^ (*d*) and TLR6^TIR^ (*e*). Conformational differences in the BCD interface, including the αC and αD helices and the CD loop of TLR2^TIR^ (boxed regions), are observed when compared with the crystal structures of TLR1, TLR2 and TLR6 TIR domains.

In the cell, TLR2 signals as a heterodimer with TLR1 or TLR6 (Jin *et al.*, 2007 ▸; Kang *et al.*, 2009 ▸) or possibly as a homodimer (Cole *et al.*, 2010 ▸; Kang *et al.*, 2009 ▸). Our study suggests that TLR2^TIR^ can nucleate the formation of MyD88^TIR^ to form higher-order assemblies, whereas TLR1^TIR^ and TLR6^TIR^ cannot. Through comparative analysis of the crystal structures of TLR1, TLR2 and TLR6 TIR domains, we observed that the BB loop and αB helix regions of TLR1^TIR^ (PDB entry 1fyv; Xu *et al.*, 2000 ▸) and TLR6^TIR^ (PDB entry 4om7; Jang & Park, 2014 ▸) share conformational similarities with TLR2^TIR^ (PDB entry 1fyw; Xu *et al.*, 2000 ▸). However, differences in the αC and αD helices and the CD-loop region between TLR1^TIR^ and TLR6^TIR^ were identified, leading us to speculate that these variations may affect the interstrand interactions that are critical for the higher-order assembly of MyD88^TIR^. Using *AlphaFold*2 *multimer* predictions (Zhu *et al.*, 2023 ▸), we modeled the recruitment of a MyD88^TIR^ molecule prompted by TLR2^TIR^, TLR1^TIR^ and TLR6^TIR^ homodimers. Our predictions suggest that TLR2^TIR^ homodimers could form a complex compatible with MyD88^TIR^ recruitment (Fig. 8 ▸; Supplementary Fig. S2). By contrast, TLR6^TIR^ and TLR1^TIR^ homodimers failed to produce an orientation compatible with MyD88^TIR^ assembly, suggesting that they cannot serve as a template for MyD88^TIR^ recruitment (Supplementary Fig. S2). Furthermore, *AlphaFold*2 predictions with multiple copies of MyD88^TIR^ suggest that TLR2^TIR^ could promote a unidirectional assembly mechanism similar to that facilitated by MAL^TIR^ (Fig. 9 ▸). Our predictions indicate that while TLR2^TIR^ is structurally similar to ‘signaling-active’ MyD88^TIR^, it presents conformational discrepancies with the crystal structures of TLR1^TIR^, TLR2^TIR^ and TLR6^TIR^ at the BCD interface. As MyD88^TIR^ assemblies and our predicted TLR2^TIR^ homodimer both self-associate through the BCD interface, these conformational differences may underlie the unique ability of TLR2^TIR^, as opposed to TLR1^TIR^ or TLR6^TIR^, to seed MyD88^TIR^ assembly (Fig. 9 ▸).

While MAL^TIR^ can more readily nucleate MyD88^TIR^ assemblies than TLR2^TIR^, the fact that TLR2^TIR^ nucleates MyD88^TIR^ microcrystal formation provides additional evidence that TLR2^TIR^ can directly interact with MyD88^TIR^ and bypass the requirement of MAL^TIR^ in certain signaling events, as has previously been reported (Kennedy *et al.*, 2014 ▸; Cole *et al.*, 2010 ▸; Kang *et al.*, 2009 ▸). Furthermore, the fact that TLR2, but not TLR1 or TLR6, is able to nucleate MyD88^TIR^ assemblies suggests that TLR2 may facilitate interaction between TLR2/1 and TLR2/6 heterodimers and MyD88. In the case of TLR2 heterodimers, MAL could be recruited to the complex and act in conjunction with TLR2 to support the recruitment of MyD88.

## 5. Conclusions

Previous work revealed that the TIR domain of MAL nucleates the assembly of MyD88 TIR domains into crystalline arrays *in vitro*. The MicroED structure of the MAL^TIR^-mediated MyD88^TIR^ higher-order assembly was refined at 3.00 Å resolution with a data completeness of 73.7%, revealing a two-stranded arrangement of TIR domains (Clabbers *et al.*, 2021 ▸), similar to those of MAL^TIR^ self-assemblies (Ve *et al.*, 2017 ▸). We found that the TIR domain of TLR2, but not those of TLR1 or TLR6, can also nucleate the formation of MyD88^TIR^ assemblies. Further, by improving the data-collection procedures, we were able to determine a MicroED structure of TLR2^TIR^-induced MyD88^TIR^ microcrystals at a higher resolution (2.85 Å) and with higher completeness (89.2%) compared with the previous MAL^TIR^-induced MyD88^TIR^ assemblies. We also show that both MAL^TIR^ and TLR2^TIR^ nucleate MyD88^TIR^ assembly unidirectionally. This study not only underscores the specific role of TLR2^TIR^ in the nucleation of MyD88^TIR^ for higher-order assembly formation, but also highlights the potential of TLR2, in combination with MAL, to bridge interactions between various TLR dimers and MyD88, paving the way for a deeper understanding of the intricate signaling mechanisms involving TLRs.

## Supplementary Material

PDB reference: MicroED structure of TLR2 TIR-domain-induced MyD88 TIR-domain higher-order assembly, 8s78

Diffraction data.: https://doi.org/10.5281/zenodo.10722078

Supplementary Figures and Table. DOI: 10.1107/S2059798324008210/gri5001sup1.pdf

## Figures and Tables

**Table 1:** Comparison of MyD88^TIR^ MicroED data-acquisition statistics Values in parentheses are for the highest resolution shell.

	TLR2^TIR^-induced MyD88^TIR^	MAL^TIR^-induced MyD88^TIR^†
Temperature (K)	77	77
Space group	*C*121	*C*121
*a*, *b*, *c* (Å)	98.07, 30.64, 53.67	99.06, 31.01, 54.30
α, β, γ (°)	90.00, 107.81, 90.00	90.00, 107.70, 90.00
No. of crystals merged	20	18
Resolution (Å)	19.34–2.85 (2.92–2.85)	30.54–3.00 (3.11–3.00)
*R*_merge_	0.510 (1.57)	0.46 (0.95)
*R*_meas_	0.525 (1.68)	Not reported
*R*_p.i.m._	0.127 (0.53)	Not reported
Mean *I*/σ(*I*)	4.4 (1.1)	4.8 (1.8)
CC_1/2_	0.96 (0.31)	0.95 (0.43)
Completeness (%)	89.2 (85.3)	73.7 (53.7)
Multiplicity	14.1 (8.2)	12.2 (6.0)
Observed reflections		
Unique	3272	2436
Total	46125	29719

† Taken from Clabbers *et al.* (2021 ▸).

**Table 2:** Comparison of MyD88^TIR^ MicroED refinement statistics

	TLR2^TIR^-induced MyD88^TIR^	MAL^TIR^-induced MyD88^TIR^†
Refinement program	*phenix.refine*	*phenix.refine*
Resolution (Å)	19.34–2.85	30.54–3.00
No. of reflections used for refinement	3253	2436
*R*_work_/*R*_free_	0.256/0.267	0.223/0.280
Mean *B* factor (Å^2^)	64.46	52.01
R.m.s. deviations		
Bond lengths (Å)	0.003	0.005
Bond angles (°)	0.62	0.52
Ramachandran plot		
Favored (%)	96.32	97.79
Allowed (%)	3.68	2.21
Outliers (%)	0.00	0.00
Clashscore	11.37	4.38
Rotamer outliers (%)	0.00	0.00

† Taken from Clabbers *et al.* (2021 ▸).
