# Supplementary material for: Microcrystal electron diffraction structure of Toll-like receptor 2 TIR-domain-nucleated MyD88 TIR-domain higher-order assembly
Source: Acta Crystallogr D Struct Biol. 2024 Sep 4;80(Pt 9):699–712. doi: 10.1107/S2059798324008210 (PMC11394124; doi:10.1107/S2059798324008210)
Supplement: Supplementary file 1 [file d-80-00699-sup1.pdf]

STRUCTURAL
BIOLOGY

Volume 80 (2024)

Supporting information for article:

Microcrystal electron diffraction structure of Toll-like receptor 2 TIR-domain-nucleated MyD88 TIR-domain higher-order assembly

Y. Li, L. C. Pacoste, W. Gu, S. Thygesen, K. Stacey, T. Ve, B. Kobe, H. Xu and J. D. Nanson

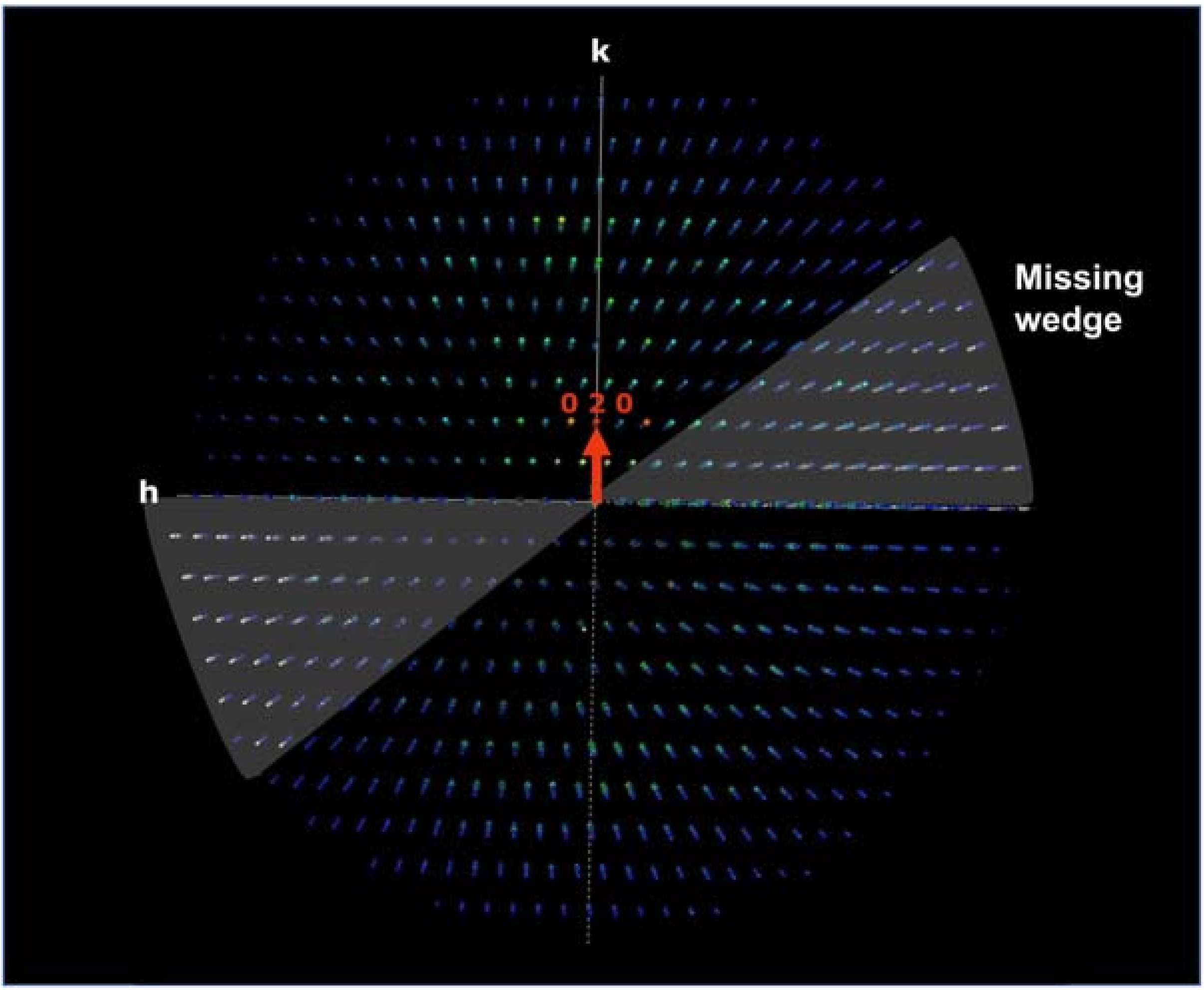

Figure S1 Three-dimensional representation of the MicroED data, highlighting the missing data wedge (white) and the reflection corresponding to the (020) planes.

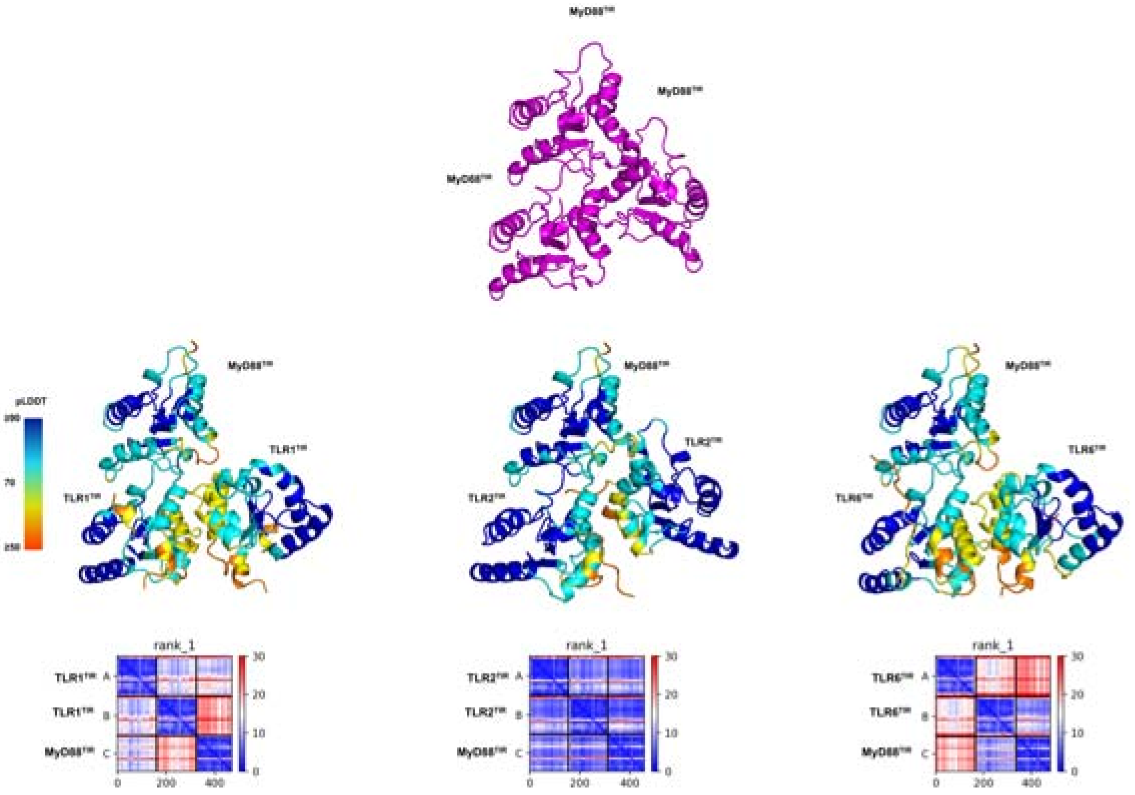

Figure S2 AlphaFold2 modelling of TLR TIR domain homodimers recruiting MyD88^{TIR}, with associated PAE plots for the top ranked models. Alphafold2 predictions of a single molecule of MyD88^{TIR} with two molecules of either TLR1^{TIR}, TLR2^{TIR}, or TLR6^{TIR}, coloured by the confidence metric (pLDDT). Only TLR2^{TIR} is predicted to form a homodimer that could serve as a template for the nucleation of MyD88^{TIR} assemblies (PDB ID: 7BEQ; magenta). Within PAE plots, chains A and B correspond to predicted TLR TIR subunits and chain C corresponds to predicted MyD88^{TIR} subunits.

Table S1 RMSD values of structural alignments shown in Figures 7 and 9.

**Aligned model**	**PDB ID**	**RMSD (Å)**	**No. Cα atoms**
Aligned to $TLR2^{TIR}$-induced $MyD88^{TIR}$ MicroED structure	8S78	-	-
MAL^{TIR}-induced $MyD88^{TIR}$ MicroED structure	7BEQ	0.35	129
$MyD88^{TIR}$ X-ray crystal structure	4EO7	0.99	94
$MyD88^{TIR}$ NMR structure	2Z5V	1.80	96
Aligned to MAL^{TIR}-induced $MyD88^{TIR}$ MicroED structure	7BEQ	-	-
$TLR2^{TIR}$ X-ray crystal structure	1FYW	1.27	80
$TLR2^{TIR}$ from AlphaFold2 homodimer	-	0.97	82
Aligned to $TLR2^{TIR}$ X-ray crystal structure	1FYW	-	-
$TLR1^{TIR}$ X-ray crystal structure	1FYV	0.79	103
$TLR6^{TIR}$ X-ray crystal structure	4OM7	1.04	106
Aligned to $TLR2^{TIR}$ from AlphaFold2 homodimer	-	-	-
$TLR1^{TIR}$ X-ray crystal structure	1FYV	0.90	95
$TLR6^{TIR}$ X-ray crystal structure	4OM7	1.25	116
